# Metformin Inhibits the Development of Hypopharyngeal Squamous Cell Carcinoma through Circ_0003214-Mediated MiR-489-3p-ADAM10 Pathway

**DOI:** 10.1155/2021/2265475

**Published:** 2021-07-13

**Authors:** Xiaoqiang Chen, Chen Li, Wei Chen, Shuchun Lin, Xuehan Yi, Qin Lin, Hao Xu, Desheng Wang

**Affiliations:** Department of Otolaryngology, Fujian Medical University Union Hospital, Fuzhou 350001, Fujian, China

## Abstract

**Purpose:**

This study aims to explore the function of metformin in hypopharyngeal squamous cell carcinoma (HSCC) and the underlying mechanism.

**Methods:**

Cell viability, colony formation, cell apoptosis, and cell cycle were investigated using cell counting kit-8 assay, colony formation, and flow cytometry assay. Gene expression was detected by quantitative real-time polymerase chain reaction and western blot. The target relationship was validated by dual-luciferase reporter assay or RNA immunoprecipitation assay. An animal study was implemented to clarify the effect of metformin *in vivo*.

**Results:**

Metformin suppressed HSCC cell viability and colony formation ability and induced cell cycle arrest and apoptosis, and circ_0003214 overexpression weakened these effects. Circ_0003214 regulated A disintegrin and metalloproteinase domain-containing protein 10 (ADAM10) expression via targeting miR-489-3p. Besides, miR-489-3p restoration reversed the role of circ_0003214, and ADAM10 knockdown reversed miR-489-3p inhibition-mediated effect. Moreover, metformin blocked tumor growth via the circ_0003214-miR-489-3p-ADAM10 axis *in vivo*.

**Conclusion:**

Metformin inhibits HSCC progression through the circ_0003214/miR-489-3p/ADAM10 pathway.

## 1. Introduction

Hypopharyngeal squamous cell carcinoma (HSCC) is one of the well-known malignant tumors of head and neck cancer, accounting for about 5% of head and neck squamous cell carcinoma [1, 2]. The early clinical symptoms of HSCC patients are not very obvious because of anatomical structure and submucosal filtration, and about 60–85% of HSCC patients are diagnosed at stages III-IV [3, 4]. Despite the progression of surgical procedures and adjuvant treatment, the overall survival rate of patients within five years is relatively low due to the local or systemic metastasis [5–7]. In order to improve the survival status of patients, effective drug treatment for HSCC has become an urgent problem to be solved.

Metformin is a biguanide oral hypoglycemic agent, which is a first-line agent for the treatment of type 2 diabetes caused by endocrine and metabolic abnormalities [8]. Accumulating studies have discovered that the use of metformin can significantly reduce the development of various tumors in diabetic patients [9, 10]. The antitumor effects of metformin are complex and mysterious in mechanism. Studies showed that metformin regulated the levels of systemic insulin or insulin-like growth factor to suppress hepatic gluconeogenesis, thus acting on tumor cell growth [11]. Besides, metformin blocked the metabolic activities of tumor cells [11, 12]. For example, metformin inhibited glioblastomas tumorigenesis and induced tumor cell cycle arrest, autophagy, and apoptosis [13]. Metformin could enhance the therapeutic effect of cisplatin in combination therapy, thus blocking the development of triple-negative breast cancer [14]. However, the effects of metformin on HSCC progression are still not fully uncovered.

Interestingly, the involvement of noncoding RNAs (ncRNAs) in the regulation of metformin has been documented in numerous diseases and cancers [15, 16], suggesting that special ncRNAs may be targets of metformin. Among these ncRNAs, circular RNAs (circRNAs) are a special type with a unique loop-closed structure and produced by “back-splicing” [17]. The discovery of circRNA function on cancer progression broadens the insights to understand cancer pathogenesis. CircRNAs are more stable than linear molecules and thus regarded as emerging biomarkers in the diagnosis and treatment of cancers [17, 18]. A previous study identified numerous circRNAs that were aberrantly regulated in HSCC tissues, and circ_0003214 was shown to be upregulated in tumor tissues [19]. However, the function of circ_0003214 in HSCC remains unclear.

CircRNAs can function as molecular sponges of downstream microRNAs (miRNAs) and thus regulate the expression of their downstream target genes [20]. The circRNA-miRNA-mRNA regulatory network was implicated in cancer development, which was widely proposed to illustrate the functional mechanism of circRNA in cancer progression [20].

In this study, we determined the effects of metformin on tumorigenesis and cell development of HSCC and characterized circ_0003214 as a target of metformin. Besides, we constructed the circ_0003214-miR-489-3p-ADAM10 regulatory network to explain the potential mechanism of circ_0003214 in metformin-mediated inhibition on HSCC progression. The aim of this paper was to understand the mechanism of metformin function in HSCC through a circRNA-dependent mode.

## 2. Materials and Methods

### 2.1. Tissues

Patients with HSCC were recruited from Fujian Medical University Union Hospital from May 2016 to July 2019. After signing the written informed consent, tumor tissues (*n* = 32) and matched normal tissues (*n* = 32) were excised by surgical excision. Then, these tissues were frozen in liquid nitrogen and preserved at −80°C condition until use. The study was implemented with the approval of the Ethics Committee of Fujian Medical University Union Hospital. Correlation between circ_0003214 expression and clinicopathologic features (such as gender, age, TNM stage, and lymph node metastasis) in patients with HSCC is presented in Table 1.

### 2.2. Cell Lines

Human HSCC cells (FaDu and Detroit 562) were provided by Procell Co., Ltd. (Wuhan, China) and maintained in matched dedicated medium (minimum essential medium containing 10% fetal bovine serum) at an incubator setting as 37°C, containing 5% CO_2_.

### 2.3. Metformin Treatment

To monitor dose effects, FaDu and Detroit 562 cells were treated with different concentrations of metformin (0, 2, 4, 6, 8, and 10 mM) (Sigma-Aldrich, St. Louis, MO, USA) for 48 h. To monitor time effects, FaDu and Detroit 562 cells were administered with 8 mM metformin for different times (0, 12, 24, 36, and 48 h). The functional experiments were performed in FaDu and Detroit 562 cells treated with 8 mM metformin for 48 h.

### 2.4. Cell Transfection

Circ_0003214 sequence fragment was cloned into pLC5-ciR vector for circ_0003214 overexpression by Geneseed Co., Ltd. (Guangzhou, China), and the fusion vector was named circ_0003214, with empty vector (vector) as a control. MiR-489-3p mimic (miR-489-3p), miR-489-3p inhibitor (anti-miR-489-3p), and matched negative control (miR-NC or anti-miR-NC) were obtained from Ribobio (Guangzhou, China) for miR-489-3p overexpression or inhibition. Besides, we used small interference RNA (siRNA) to mediate ADAM10 downregulation (si-ADAM10), which was synthesized by Ribobio, with si-NC as a negative control. FaDu and Detroit 562 cells were transfected alone or cotransfected with the abovementioned oligonucleotides or plasmids using the Lipofectamine 3000 reagent (Invitrogen, Carlsbad, CA, USA).

### 2.5. Quantitative Real-Time Polymerase Chain Reaction (qRT-PCR)

Total RNA was isolated using the RNAiso Plus (Takara, Dalian, China) and then examined using NanoDrop 2000 (Thermo Fisher Scientific, Waltham, MA, USA). For cDNA synthesis of circ_0003214 and ADAM10; a reverse transcription kit (Takara) was used for reverse transcription, followed by a qRT-PCR amplification reaction using the SYBR Premix Ex Taq (Takara). For cDNA synthesis of miR-489-3p, the miRNA First-Strand Synthesis Kit (Clontech, Mountain View, CA, USA) was used, followed by a qRT-PCR amplification reaction using the miRNA qRT-PCR TB Green Kit (Clontech). GAPDH or U6 was set as an internal reference, and the expression was normalized and calculated using the 2^−ΔΔct^ method. This experiment involved three repetitions. The primers used were listed as follows: circ_0003214, F, 5'-ACTACGATCATCAATGCTGTGG-3' and R, 5'-GGGCAAAGTCAAAGATCTGGT-3'; GAPDH, F, 5'-AAGGTCATCCCTGAGCTGAAC-3' and R, 5'-TGAAGTCAGAGGAGACCACC-3'; miR-489-3p, F, 5'-GGGGTGACATCACATATAC-3' and R, 5'-CAGTGCGTGTCGTGGAGT-3'; U6, F, 5'-CTCGCTTCGGCAGCACATATACT-3' and R, 5'-ACGCTTCACGAATTTGCGTGTC-3'; ADAM10, F, 5'-AAGAAGCTTCCCACAAGGCA-3' and R, 5'-TGTGTACGCAGAGTATCTAACTGG-3'.

### 2.6. Cell Counting Kit-8 (CCK-8) Assay

To monitor dose effects, FaDu and Detroit 562 cells were treated with different concentrations of metformin (0, 2, 4, 6, 8, and 10 mM) and loaded into 96-well plates (Corning Costar, Corning, NY, USA) at a density of 2 × 10^3^ cells/well. After 48 h, 10 *µ*L CCK-8 reagent was added into each well, incubating for 2 h, and then the optical density (OD) value at 450 nm was measured using Multiskan Ascent (Thermo Fisher Scientific). To monitor time effects, FaDu and Detroit 562 cells were treated with 8 mM metformin and loaded into 96-well plates (Corning Costar); 10 *µ*L CCK-8 reagent was added into each well at the indicated time points (0, 12, 24, 36, and 48 h), incubating for another 2 h. The optical density (OD) value at 450 nm was measured using Multiskan Ascent (Thermo Fisher Scientific). This experiment involved three repetitions.

### 2.7. Colony Formation Assay

Metformin-treated cells with different transfection were planted into 6-well plates (Corning Costar) at the density of 1.5 × 10^2^ cells/well and cultured for 8 days. After that, the cell medium was discarded, and the colonies were immobilized with methanol and stained with crystal violet (Beyotime, Shanghai, China). After photographing, the number of colonies was calculated under a microscope (Olympus, Tokyo, Japan). This experiment involved three repetitions.

### 2.8. Flow Cytometry Assay

The Annexin V-FITC Apoptosis Detection Kit (Solarbio, Beijing, China) was used to examine cell apoptosis. In brief, cells were collected at 48 h after transfection and then washed with cooled phosphate-buffered saline (PBS). Next, cells were suspended in prescribed binding buffer, and Annexin V-FITC and Propidium Iodide (PI) solution were consecutively added to stain cells at room temperature in the dark following the guidelines. The apoptotic cells were monitored using Attune NxT Flow Cytometer (Invitrogen). This experiment involved three repetitions.

For cell cycle analysis, cells were collected at 48 h after transfection and then washed with cooled PBS. After collection by centrifugation, cells were fixed in 70% ethyl alcohol at 4°C overnight. Afterwards, cells were washed with PBS again and incubated with a PI solution containing RNase A (Solarbio) at room temperature in the dark. Cells in different stages were analyzed using Attune NxT Flow Cytometer (Invitrogen). This experiment involved three repetitions.

### 2.9. Target Prediction

The potential targets of circ_0003214 or miR-489-3p were predicted by the bioinformatics tool starBase v3.0 (http://starbase.sysu.edu.cn/), which provided the targeting sites between their sequence fragments.

### 2.10. Dual-Luciferase Reporter Assay

Circ_0003214 fragment containing the putative miR-489-3p targeting site was amplified. To construct the mutant circ_0003214 fragment, the miR-489-3p targeting site (UGAUGUCA) within the circ_0003214 fragment was changed to UCAUCUGA.

The wild type and mutant type of circ_0003214 fragment were constructed into pmirGLO vector (Promega, Madison, WI, USA), named circ_0003214 WT or circ_0003214 MUT. Similarly, fusion plasmid ADAM10 3'UTR-WT or ADAM10 3'UTR-MUT was also constructed. For luciferase reporter assay, FaDu and Detroit 562 cells were transfected with circ_0003214 WT, circ_0003214 MUT, ADAM10 3'UTR-WT or ADAM10 3'UTR-MUT, and miR-489-3p or miR-NC, respectively. At 48 h after transfection, the Dual-Luciferase Assay System (Promega) was used to measure the luciferase activity. This experiment involved three repetitions.

### 2.11. RNA Immunoprecipitation (RIP) Assay

Magna RIP RNA-Binding Protein Immunoprecipitation Kit (Millipore, Billerica, MA, USA) was applied for RIP assay according to the protocol. Human argonaute-2 (Ago2, Millipore) was used for experimental analysis and mouse.

Immunoglobulin G (IgG, Millipore) was used as a negative control; the precipitated RNA was isolated and subjected to qRT-PCR as mentioned above. This experiment involved three repetitions.

### 2.12. Western Blot

Western blot analysis was conducted to evaluate the expression of ADAM10 at the protein level. Total protein was extracted using the RIPA lysis buffer (Beyotime) in accordance with the instructions and then examined using a BCA kit (Beyotime). After separation by 10% SDS-PAGE, the proteins were blotted onto PVDF membranes (Bio-Rad, Hercules, CA, USA), followed by the blockage in 5% skim milk. Then, the membranes were incubated with the primary antibodies, including anti-ADAM10 (ab124695, Abcam, Cambridge, MA, USA) and anti-GAPDH (ab9485, Abcam). Subsequently, the membranes were incubated with HRP-labeled goat anti-rabbit antibody (ab205718, Abcam). The protein signals were emerged using the enhanced chemiluminescent reagent (Beyotime), and the ImageJ 1.48u software (NIH, Bethesda, MA, USA) was utilized for protein quantification. This experiment involved three repetitions.

### 2.13. *In Vivo* Assay

A total of 15 Balb/c experimental mice (female, 6–8 weeks old, 18–20 g) were purchased from HFK Bioscience Co., Ltd. (Beijing, China) and maintained in a pathogen-free room. Circ_0003214 fragments cloned into pLC5-ciR vector were transfected into 293T cells, followed by the lentiviral package by a commercial company (Geneseed). FaDu cells were infected with lentivirus suspension containing circ_0003214 overexpression and then selected by hygromycin. FaDu cells with or without circ_0003214 overexpression were subcutaneously implanted into nude mice for tumor growth. The mice were divided into three groups (*n* = 5/group), including the control group (only FaDu cell implant), metformin group (FaDu cell implant and metformin treatment), and metformin + circ_0003214 group (FaDu cell with circ_0003214 overexpression implant and metformin treatment). When tumor nodes grew to about 60 mm^2^, metformin was used to administer nude mice by intraperitoneal injection, every three days. During tumor growth, tumor volume (length × width^2^ × 0.5) was recorded every 4 days. At the 27^th^ day after implant, all mice were killed to remove tumor tissues for the following analyses. The mice were anesthetized using pentobarbitone and then sacrificed via cervical dislocation. The animal study was carried out with the approval of the Animal Care and Use Committee of Fujian Medical University Union Hospital.

### 2.14. Statistical Analysis

All experiments contained at least three repetitions. All data were analyzed by GraphPad Prism 5.0 (GraphPad; La Jolla, CA, USA). Measurement data were shown in the form of mean ± standard deviation (SD). The comparisons between two groups were analyzed by unpaired Student's *t*-test, and the comparisons among multiple groups were performed by one-way analysis of variance (ANOVA). A value of *P* < 0.05 was considered to be statistically significant.

## 3. Results and Discussion

### 3.1. Result

#### 3.1.1. Metformin Weakened HSCC Cell Viability

**Figure 1:**
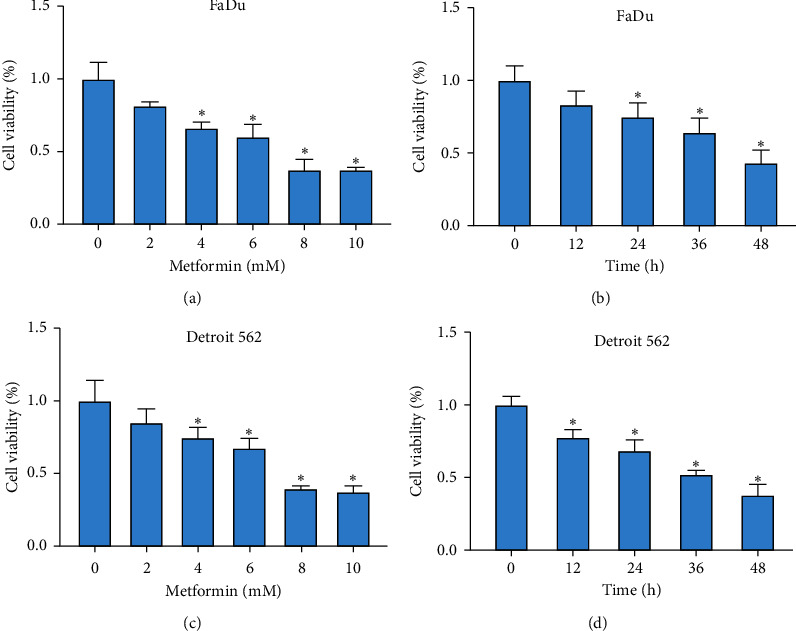
Metformin suppressed cell viability of FaDu and Detroit 562 cells. ((a) and (c)) FaDu and Detroit 562 cells were treated with different concentrations of metformin (0, 2, 4, 6, 8, and 10 mM) for 48 h and cell viability was checked using CCK-8 assay. ((b) and (d)) FaDu and Detroit 562 cells were treated with 8 mM metformin for different times (0, 12, 24, 36, and 48 h), and cell viability was checked using CCK-8 assay. ^*∗*^*P* < 0.05.

To determine the effects of metformin in HSCC cells, FaDu and Detroit 562 cells were treated with metformin at different concentrations and incubated for different times. As a result, metformin treatment resulted in a decrease of cell viability in FaDu and Detroit 562 cells in a dose-dependent manner (0, 2, 4, 6, 8, and 10 mM) (Figures 1(a) and 1(c)). Besides, FaDu and Detroit 562 cell viability was decreased after the treatment of metformin in a time-dependent manner (0, 12, 24, 36, and 48 h) (Figures 1(b) and 1(d)). According to the data, FaDu and Detroit 562 cells treated with 8 mM for 48 h were used in the following experiments.

#### 3.1.2. Circ_0003214 Overexpression Partly Abolished the Effects of Metformin

**Figure 2:**
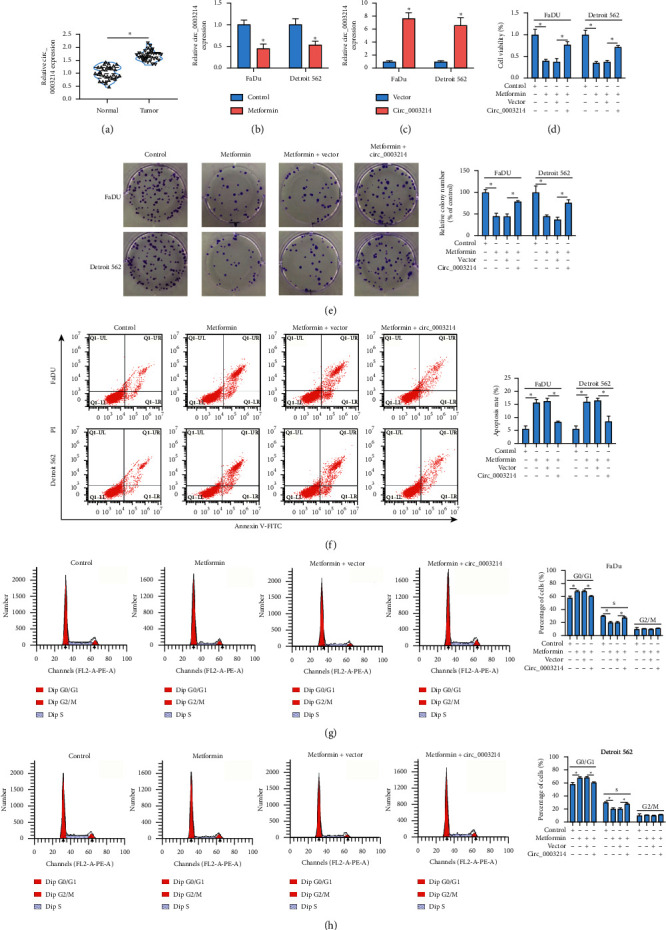
Metformin suppressed HSCC development *in vitro* by lessening circ_0003214 expression. (a) The expression of circ_0003214 in HSCC tumor tissues and normal tissues was detected by qRT-PCR. (b) The expression of circ_0003214 in metformin-treated FaDu and Detroit 562 cells was detected by qRT-PCR. (c) The efficiency of circ_0003214 overexpression in FaDu and Detroit 562 cells was checked by qRT-PCR. In metformin-treated FaDu and Detroit 562 cells transfected with circ_0003214 or vector, (d) cell viability was assessed using CCK-8 assay. (e) The ability of colony formation was determined by colony formation assay. (f, g, h) Cell apoptosis and cell cycle progression were investigated using flow cytometry assay. ^*∗*^*P* < 0.05.

The data from qRT-PCR showed that the expression of circ_0003214 was strikingly increased in HSCC tumor tissues compared with that in normal tissues (Figure 2(a)). However, the expression of circ_0003214 was significantly declined in metformin-treated FaDu and Detroit 562 cells (Figure 2(b)). Then, we constructed circ_0003214 overexpression vector and transfected it into FaDu and Detroit 562 cells to induce circ_0003214 overexpression. The data showed that circ_0003214 expression was notably enhanced in cells after circ_0003214 transfection (Figure 2(c)). Along with the overexpression of circ_0003214, metformin-suppressed cell viability was largely recovered (Figure 2(d)). Similarly, the ability of colony formation weakened by metformin treatment was also restored by circ_0003214 reintroduction (Figure 2(e)). Moreover, metformin-induced cell apoptosis was partly suppressed by circ_0003214 overexpression (Figure 2(f)). Metformin treatment also induced cell cycle arrest, while circ_0003214 reintroduction promoted cell cycle progression (Figures 2(g) and 2(h)). These data suggested that metformin treatment blocked various HSCC cell malignant behaviors, while circ_0003214 overexpression partly abolished the role of metformin and triggered these malignant behaviors.

#### 3.1.3. MiR-489-3p Was a Target of Circ_0003214

**Figure 3:**
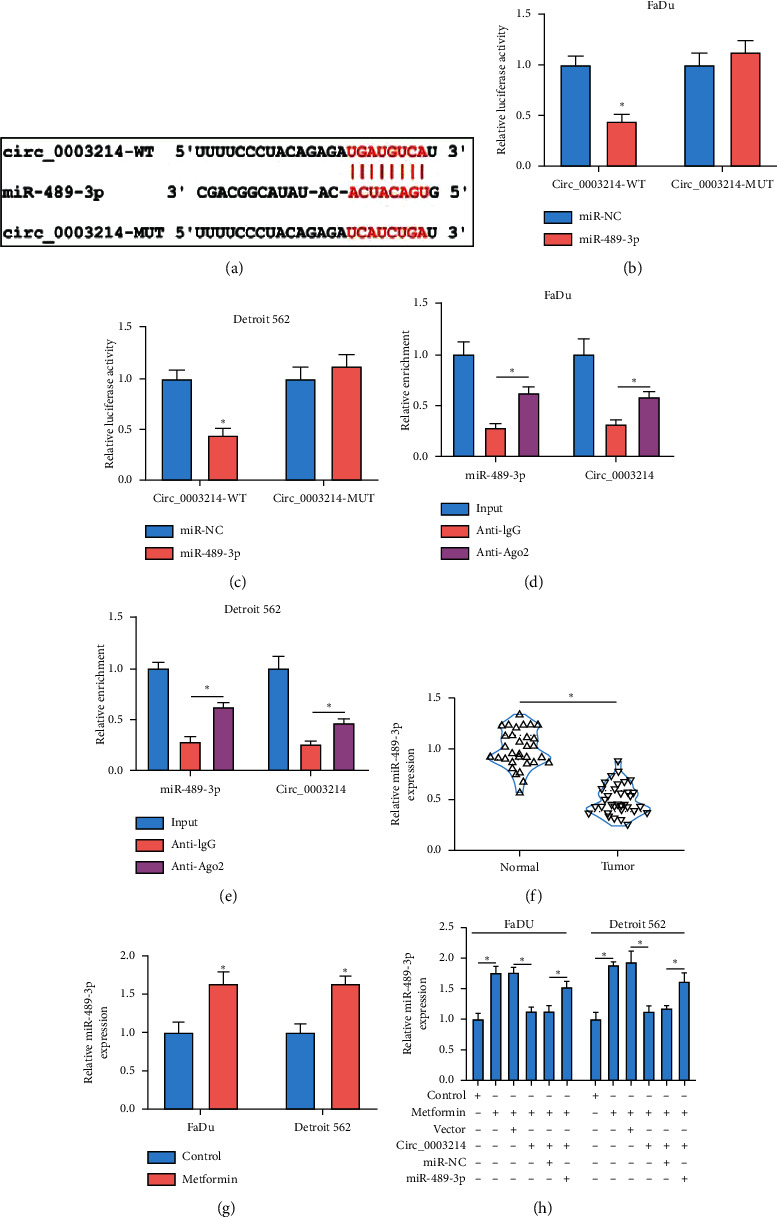
MiR-489-3p was a target of circ_0003214. (a) The target site between circ_0003214 and miR-489-3p was predicted by starBase v3.0. MiR-489-3p was targeted by circ_0003214, which was verified by ((b) and (c)) dual-luciferase reporter assay and ((d) and (e)) RIP assay. (f) The expression of miR-489-3p in HSCC tumor tissues and normal tissues was detected by qRT-PCR. (g) The expression of miR-489-3p in metformin-treated FaDu and Detroit 562 cells was detected by qRT-PCR. (h) The expression of miR-489-3p in metformin-treated FaDu and Detroit 562 cells transfected with circ_0003214, vector, circ_0003214+miR-489-3p, or circ_0003214+miR-NC was detected by qRT-PCR. ^*∗*^*P* < 0.05.

To explore the functional mechanism of circ_0003214 in metformin-treated HSCC, we ensured the target miRNAs of circ_0003214. MiR-489-3p was predicted as a potential target of circ_0003214 by starBase v3.0 because there was a special binding site between circ_0003214 and miR-489-3p sequence fragment (Figure 3(a)). Subsequent dual-luciferase reporter assay presented that the cotransfection of miR-489-3p and circ_0003214-WT could significantly reduce the luciferase activity in FaDu and Detroit 562 cells (Figures 3(b) and 3(c)). Further RIP assay showed that both circ_0003214 and miR-489-3p could be abundantly detected in the Anti-Ago2 group compared with those in the Anti-IgG group (Figures 3(d) and 3(e)). The supporting evidence ensured that miR-489-3p was a target of circ_0003214. The expression of miR-489-3p was lower in HSCC tumor tissues compared to normal tissues (Figure 3(f)), while the expression of miR-489-3p was strikingly promoted in metformin-treated FaDu and Detroit 562 cells (Figure 3(g)). Besides, the expression of miR-489-3p promoted in metformin-treated FaDu and Detroit 562 cells was weakened by circ_0003214 reintroduction, while additional miR-489-3p mimic transfection significantly recovered miR-489-3p expression (Figure 3(h)), hinting that miR-489-3p might be involved in the metformin-circ_0003214 regulatory pathway.

#### 3.1.4. Circ_0003214 Overexpression Abolished the Effects of Metformin by Targeting MiR-489-3p

**Figure 4:**
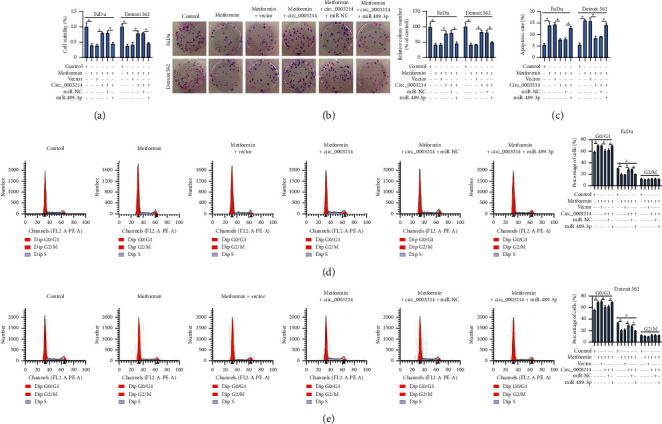
Circ_0003214 overexpression abolished the effects of metformin by targeting miR-489-3p. In metformin-treated FaDu and Detroit 562 cells transfected with circ_0003214, vector, circ_0003214 + miR-489-3p or circ_0003214 + miR-NC, ((a) and (b)) cell viability and colony formation were assessed using CCK-8 assay and colony formation assay, respectively. (c–e) Cell apoptosis and cell cycle progression were determined by flow cytometry assay. ^*∗*^*P* < 0.05.

Next, we transfected circ_0003214 alone or circ_0003214 + miR-489-3p into metformin-treated FaDu and Detroit 562 cells to observe the effects. The data showed that metformin-blocked cell viability and colony formation ability were recovered by circ_0003214 transfection, while combined circ_0003214 + miR-489-3p transfection largely impaired cell viability and colony formation ability (Figures 4(a) and 4(b)). The number of apoptotic cells was suppressed in metformin-treated cells with circ_0003214 transfection but partly promoted in metformin-treated cells with circ_0003214 + miR-489-3p transfection compared to circ_0003214 + miR-NC transfection (Figure 4(c)). Moreover, metformin-induced cell cycle arrest was lessened by circ_0003214 reintroduction but recovered by additional miR-489-3p restoration (Figures 4(d) and 4(e)). The data indicated that circ_0003214 promoted HSCC cell growth by weakening miR-489-3p expression in metformin-treated HSCC cells.

#### 3.1.5. ADAM10 Was a Target of MiR-489-3p

**Figure 5:**
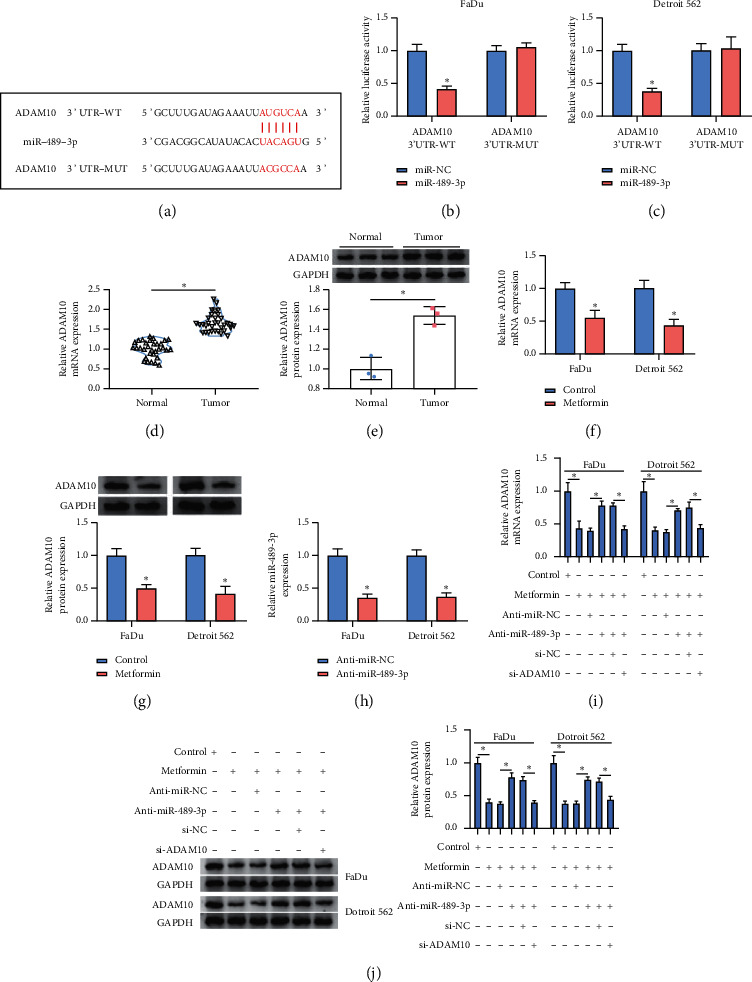
ADAM10 was a target of miR-489-3p. (a) ADAM10 was predicted as a target of miR-489-3p by starBase v3.0. (b, c)) The predicted target relationship between miR-489-3p and ADAM10 was validated by a dual-luciferase reporter assay. (d, e)) The expression of ADAM10 in HSCC tumor tissues and normal tissues was detected by qRT-PCR and western blot. (f, g)) The expression of ADAM10 in metformin-treated FaDu and Detroit 562 cells was detected by qRT-PCR and western blot. (h) The efficiency of miR-489-3p inhibitor was checked using qRT-PCR. (i, j)) The expression of ADAM10 in metformin-treated FaDu and Detroit 562 cells transfected with anti-miR-489-3p, anti-miR-NC, anti-miR-489-3p + si-ADAM10, or anti-miR-489-3p + si-NC was detected by qRT-PCR and western blot. ^*∗*^*P* < 0.05.

To further explore the regulatory pathway of circ_0003214, the potential targets mRNAs of miR-489-3p were also determined. As displayed in Figure 5(a), miR-489-3p might bind to ADAM10 3'UTR through a special binding site, hinting that ADAM10 might be a target of miR-489-3p (Figure 5(a)). Then, dual-luciferase reporter assay verified this prediction and manifested that the luciferase activity was strikingly decreased in FaDu and Detroit 562 cells with miR-489-3p and ADAM10 3'UTR-WT transfection (Figures 5(b) and 5(c)). We next examined the expression of ADAM10 and found that ADAM10 was highly expressed in HSCC tumor tissues compared to normal tissues (Figures 5(d) and 5(e)). Besides, the expression of ADAM10 was significantly declined in metformin-treated FaDu and Detroit 562 cells (Figures 5(f) and 5(g)). For the following experiments, the examination of anti-miR-489-3p efficiency showed that the expression of miR-489-3p was strikingly declined in FaDu and Detroit 562 cells transfected with anti-miR-489-3p (Figure 5(h)). Next, the expression of ADAM10 was impaired in metformin-treated FaDu and Detroit 562 cells but largely recovered by the transfection of anti-miR-489-3p, while additional si-ADAM10 transfection repressed the expression of ADAM10 (Figures 5(i) and 5(j)), suggesting that miR-489-3p could suppress ADAM10 expression.

#### 3.1.6. MiR-489-3p Inhibition Abolished the Effects of Metformin by Increasing ADAM10 Expression

**Figure 6:**
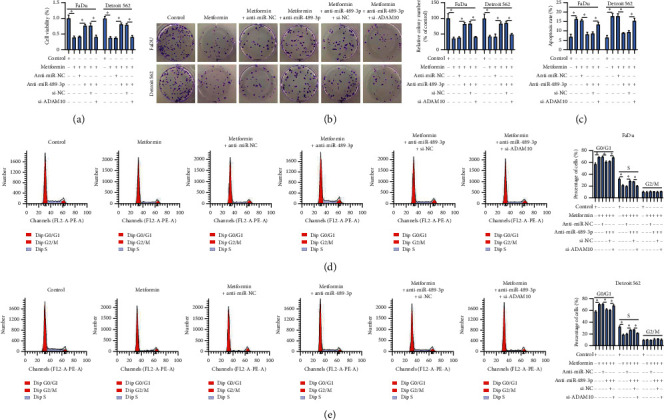
MiR-489-3p inhibition abolished the effects of metformin by mediating ADAM10. In metformin-treated FaDu and Detroit 562 cells transfected with anti-miR-489-3p, anti-miR-NC, anti-miR-489-3p + si-ADAM10, or anti-miR-489-3p + si-NC ((a) and (b)) cell viability and colony formation were investigated using CCK-8 assay and colony formation assay, respectively. (c–e) Cell apoptosis and cell cycle progression were monitored by flow cytometry assay. ^*∗*^*P* < 0.05.

Following rescue experiments were performed to determine whether miR-489-3p could suppress ADAM10 function. In detail, cell viability and colony formation ability were promoted in metformin-treated FaDu and Detroit 562 cells with anti-miR-489-3p transfection relative to anti-miR-NC transfection but impaired in cells with anti-miR-489-3p + si-ADAM10 transfection relative to anti-miR-489-3p + si-NC transfection (Figures 6(a) and 6(b)). In addition, metformin-induced cell apoptosis and cell cycle arrest were alleviated by anti-miR-489-3p transfection, while the cotransfection of anti-miR-489-3p + si-ADAM10 promoted cell apoptosis and cell cycle arrest (Figures 6(c)–6(e)). The data showed that miR-489-3p deficiency recovered the inhibitory effects of metformin on HSCC cell development by enriching ADAM10 expression.

#### 3.1.7. Circ_0003214 Upregulated ADAM10 by Suppressing miR-489-3p in Metformin-Treated FaDu and Detroit 562 Cells

**Figure 7:**
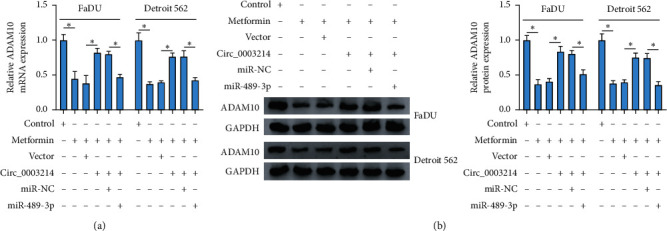
Circ_0003214 upregulated ADAM10 by targeting miR-489-3p. (a, b)) The expression of ADAM10 in metformin-treated FaDu and Detroit 562 cells transfected with circ_0003214, vector, circ_0003214 + miR-489-3p, or circ_0003214 + miR-NC was detected using qRT-PCR and western blot at both mRNA and protein levels. ^*∗*^*P* < 0.05.

Metformin-treated FaDu and Detroit 562 cells were transfected with circ_0003214 or circ_0003214 + miR-489-3p, with vector or circ_0003214 + miR-NC as the control, and the expression of ADAM10 was checked in these cells. The result showed that the expression of ADAM10 was elevated in metformin-treated cells transfected with circ_0003214 but repressed in cells transfected with circ_0003214 + miR-489-3p at both mRNA and protein levels (Figures 7(a) and 7(b)), suggesting that metformin blocked HSCC progression through the circ_0003214-miR-489-3p-ADAM10 regulatory pathway.

#### 3.1.8. Metformin Inhibited Tumor Growth *In Vitro* through Weakening Circ_0003214 Expression

**Figure 8:**
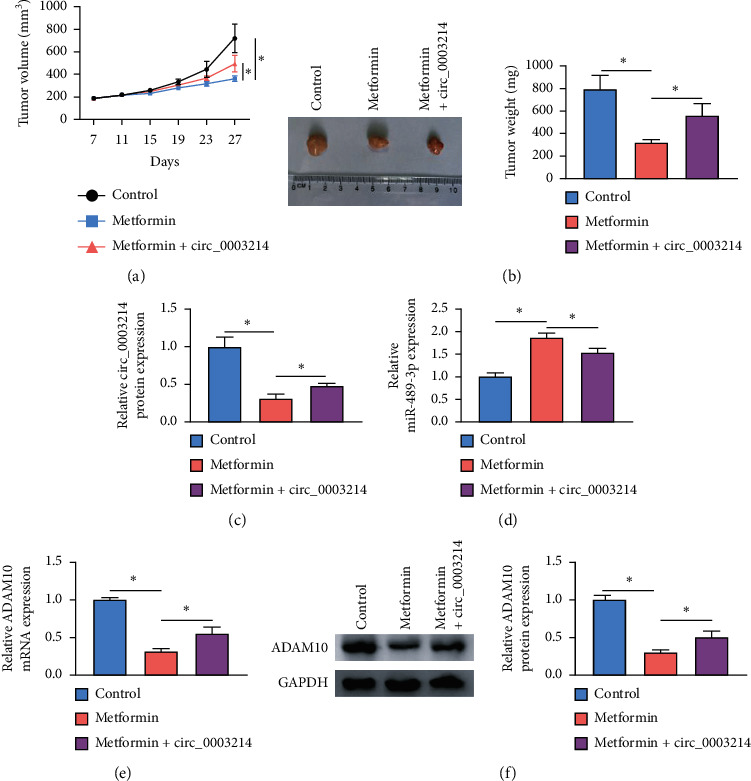
Metformin inhibited tumor growth *in vivo* by regulating the circ_0003214-miR-489-3p-ADAM10 pathway. The experimental mice were divided into three groups, including control (nontreatment), metformin (metformin treatment), and metformin + circ_0003214 (metformin treatment and circ_0003214 overexpression). (a) Tumor volume was measured every 4 days. (b) Tumor weight was measured at 27 days after injection. (c, d)) The expression of circ_0003214 and miR-489-3p in the excised tumor tissues was detected by qRT-PCR. (e, f)) The expression of ADAM10 in the excised tumor tissues was detected by qRT-PCR and western blot. ^*∗*^*P* < 0.05.

We further investigated the role of metformin *in vivo*. The result presented that metformin treatment significantly weakened tumor volume and tumor weight compared to control (nontreatment), while synchronous circ_0003214 overexpression partly estimated the effect of metformin and increased tumor volume and tumor weight (Figures 8(a) and 8(b)). Furthermore, the expression of circ_0003214, miR-489-3p, and ADAM10 was examined in the excised tumor tissues. As shown in Figures 8(c) and 8(d), the expression of circ_0003214 was impaired, while the expression of miR-489-3p was elevated in tissues from the metformin-treated group. However, the expression of circ_0003214 was enhanced, while the expression of miR-489-3p was lessened in tissues from the metformin + circ_0003214 group (Figures 8(c) and 8(d)). The expression pattern of ADAM10 in tissues from different groups was consistent with the expression pattern of circ_0003214 (Figures 8(e) and 8(f)). The data suggested that metformin suppressed tumor growth by regulating the circ_0003214-miR-489-3p-ADAM10 regulatory pathway.

### 3.2. Discussion

Metformin is a common treasure of agent, not only in the treatment of diabetes but also in antiaging management, the defense of cancers, and multiple diseases [21–23]. A previous study reported that metformin significantly inhibited the proliferation of HSCC cell line (FaDu) in a dose- and time-dependent manner [24]. Tsou et al. assessed the effects of metformin on HSCC in patients with diabetes mellitus and concluded that metformin resulted in decreased distant metastasis of HSCC and better survival outcomes [25]. Wu et al. demonstrated that metformin inhibited HSCC progression and sensitized HSCC cell to taxol and irradiation by the suppression of long ncRNA SNHG7 [26]. Besides, the antitumor effects of metformin were also presented in other cancers, such as pancreatic cancer, breast cancer, and head and neck cancer [27–29]. These data suggested that metformin suppressed the progression of diverse cancers. In our study, similarly, we showed that metformin usage repressed HSCC cell growth and induced cell apoptosis. Moreover, an animal study announced that metformin inhibited tumor growth *in vivo*. The sufficient evidence indicated that metformin prevented the development of HSCC.

Previous studies proposed that metformin blocked cancer progression by regulating the expression of ncRNAs [15, 16]. We hence speculated that metformin might also mediate the expression of special circRNAs. Following this hypothesis, we noticed that circ_0003214 was highly expressed in HSCC tissues but notably downregulated in HSCC cells with the treatment of metformin. Circ_0003214 was shown to be upregulated in HSCC tissues by RNA sequencing technology [19], hinting that circ_0003214 might be involved in HSCC development. In our data, we demonstrated that circ_0003214 participated in metformin-mediated inhibition of HSCC development, and its overexpression partly abolished the role of metformin to induce tumor cell malignant traits.

Subsequent experiments exhibited that circ_0003214 acted as a sponge to suppress miR-489-3p expression. Kikkawa et al. previously reported that miR-489-3p was poorly expressed in HSCC tumor tissues and cells, and they defined miR-489-3p as a tumor inhibitor in HSCC [30]. Furthermore, miR-489-3p deficiency activated the PAX3-MET pathway to aggravate the metastasis of osteosarcoma [31]. Likewise, miR-489-3p overexpression also impaired the proliferation and invasion of bladder cancer cells by degrading its target genes [32]. Consistent with these results, we discovered that miR-489-3p was downregulated in HSCC tissues but upregulated in metformin-treated cells. In function, miR-489-3p restoration reversed the effects of circ_0003214 overexpression, and miR-489-3p deficiency also partly abolished the role of metformin, suggesting that circ_0003214 suppressed the expression of miR-489-3p to be implicated in metformin-inhibited HSCC development.

A further study illustrated that miR-489-3p directly bound to ADAM10 3'UTR, suggesting that miR-489-3p might block the function of ADAM10. ADAM10 overexpression was reported to trigger tumor cell proliferation and migration and result in a poor prognosis of HSCC [33]. ADAM10 was also identified to be a target of miR-140-5p, and ADAM10 knockdown suppressed HSCC cell proliferation, migration, and invasion [34]. In our study, we defined that ADAM10 was involved in metformin-inhibited HSCC development through the circ_0003214-miR-489-3p network because circ_0003214 could upregulate the expression of ADAM10 by sponging miR-489-3p.

## 4. Conclusion

In conclusion, metformin inhibited HSCC cell viability, colony formation, and cell cycle progression and blocked tumor growth *in vivo*. Circ_0003214 was implicated in metformin-mediated inhibition of HSCC progression through the circ_0003214-miR-489-3p-ADAM10 regulatory network. Our study enriched the functional mechanisms of metformin in HSCC and first explored the function of circ_0003214 in HSCC, aiming to provide a better strategy for HSCC treatment.

## Figures and Tables

**Table 1:** Correlation between the expression of circ_0003214 and clinicopathologic features in patients with hypopharyngeal squamous cell carcinoma.

Parameters	*N* = 32	Circ_0003214 expression		*p* value
		High *N* = 17	Low *N* = 15	
Age, years				
<60	12	5	7	0.314
≥60	20	12	8	

Sex				
Male	17	9	8	0.982
Female	15	8	7	

TNM stage				
I-II	21	7	14	0.002^*∗*^
III	11	10	1	

Lymph node metastasis				
No	24	10	14	0.024^*∗*^
Yes	8	7	1	

^*∗*^ *P* < 0.05.

## Data Availability

The data used to support the findings of this study are available from the corresponding author upon request.
